# Supplementary material for: EpiBeds: Data informed modelling of the COVID-19 hospital burden in England
Source: PLoS Comput Biol. 2022 Sep 6;18(9):e1010406. doi: 10.1371/journal.pcbi.1010406 (PMC9481171; doi:10.1371/journal.pcbi.1010406)
Supplement: S1 Supplementary Material — Extra figures supporting the narrative. Additional results detailing the input parameters used for the performance evaluation. (DOCX) [file pcbi.1010406.s001.docx]

**Supplementary Material - EpiBeds: Data informed modelling of the COVID-19 hospital burden in England**

**Christopher E. Overton^*,1,2,3,16^, Lorenzo Pellis^*,1,3,14^, Helena B. Stage^1,4,5^, Francesca Scarabel^1,3^, Joshua Burton^7^, Christophe Fraser^9,11,12^, Ian Hall^1,2,3,14,15^, Thomas A. House^1,2.3,7,14^, Chris Jewell^8^, Anel Nurtay^9^, Filippo Pagani^1,6^, Katrina A. Lythgoe^9,10^**

** These authors contributed equally.*

*^1^ Department of Mathematics, University of Manchester, UK.*

*^2^ Clinical Data Science Unit, Manchester University NHS Foundation Trust, UK.*

*^3^ Joint UNIversities Pandemic and Epidemiological Research,* [*https://maths.org/juniper/*](https://maths.org/juniper/)*.*

*^4^ The Humboldt University of Berlin, Germany.*

*^5^ The University of Potsdam, Germany.*

*^6^ MRC Biostatistics Unit, University of Cambridge, UK.*

*^7^ Division of Informatics, Imaging and Data Sciences, Faculty of Biology Medicine and Health, University of Manchester, UK.*

*^8^ CHICAS, Lancaster Medical School, Lancaster University, UK.*

*^9^ Big Data Institute, Nuffield Department of Medicine, University of Oxford, UK.*

*^10^ Department of Biology, University of Oxford, UK.*

*^11^ Wellcome Centre for Human Genetics, Nuffield Department of Medicine, NIHR Biomedical Research Centre, University of Oxford, UK.*

*^12^ Wellcome Sanger Institute, Cambridge, UK.*

*^13^ IBM Research, Hartree Centre, Daresbury, UK.*

*^14^ Alan Turing Institute, UK.*

*^15^ Emergency Preparedness, Health Protection Division, UK Health Security Agency, UK.*

*^16^ Infectious Disease Modelling, All Hazards Intelligence, UK Health Security Agency, UK.*

**SM.1 Supplementary Methods**

*SM.1.1 Data sets*

*SM.1.1.1 CHESS/SARI*

CHESS (COVID-19 Hospitalisations in England Surveillance System) and SARI (Severe Acute Respiratory Illness), which replaced CHESS, record patient-level data detailing the date of each event in their hospital pathway, such as time of hospital admission and discharge, time of ICU admission and discharge, and time of death in hospital. This provides detail into how long individuals spend in hospital and the type of pathways they take. We also used these data to estimate a prior distribution for the probability that patients die in ICU, since ICU patient coverage is very high. However, CHESS/SARI is heavily biased towards patients that require critical care and therefore do not provide reliable indicators of the overall hospital burden.

*SM.1.1.2 SITREP*

SITREP (NHS situation report) contains the number of COVID-19 positive patients in various bed types in each hospital. Data includes the number of admissions each day who had a positive test before admission, the daily number of inpatients who have been newly diagnosed with COVID-19, the number of COVID-19 patients in any hospital bed and the number of COVID-19 patients in ICU beds. Unlike the CHESS data, this is a mostly complete data set.

In the early days of the pandemic, data quality issues were evident in the data submitted between different hospital trusts. To mitigate these issues, in the first wave, we only included individuals who tested positive for COVID-19 after admission. This is justified since there was very little testing outside of hospitals during this time. By the second wave, these early data quality issues had been solved enabling us to include individuals testing positive prior to hospital admission. This resulted in the modelling prior to 15^th^ September 2020 having fewer hospital admissions during the first wave than the post 1^st^ August 2020 modelling. Despite the potential quality issues in these data, the SITREP provides the most reliable time-series for hospitalisation data in England.

*SM.1.1.3 CPNS*

CPNS (COVID-19 Patient Notification System) is a complete line list of all COVID-19 positive individuals, from which we determined the number of COVID-19 hospital deaths each day. Since CPNS records the deaths of all individuals who tested positive for COVID-19 within the previous 28 days, some of these individuals may have died of other causes. However, these figures are very close to the number of deaths with COVID-19 listed as a cause of death, with 100,000 reported by the Office of National Statistics COVID-19 Infection Survey (ONS CIS) [1] compared to 95000 in the CPNS [2], as of 18^th^ January 2021.

*SM.1.2 Estimating delay distributions*

Delay distributions describe the distribution of times between subsequent events. We determined the delay distribution that maximised the likelihood of the observed event dates by conditioning on the time of the first event and looking forwards to the probability of the second event occurring on the observed date. Because we were collecting data during an ongoing outbreak, the sample was truncated, meaning we are more likely to observe short delays. To account for this, we condition the likelihood function against the second event being observed before the truncation date [3].

Let *E*_1_ denote the first event and *E*_2_ denote the second event. The truncation date will be denoted by *T*. For a sample *x* with *E*_1_ *= e*_1_ and *E*_2_ *= e*_2_, the corresponding likelihood function is [3]


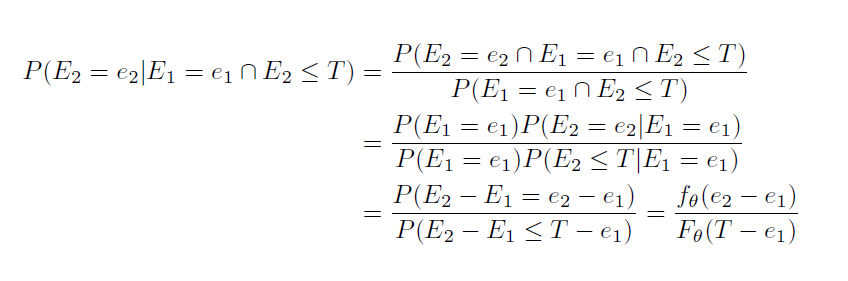


where *f_θ_(.)* is the probability density function and *F_θ_(.)* is the cumulative density function of the delay between events *E*_1_ and *E*_2_, parameterised by *θ*.

Since this likelihood function conditions on both events being observed before the truncation date, we condition the sample for each delay to only include cases for which both events have been observed. This removes censoring issues present in the CHESS data, and the truncation correction should correct for the fewer long delays observed. When fitting the delays, we fit the first and second waves independently, to account for changes in these delays. For the first wave, we used data from 1^st^ March 2020 and generated estimates using data available up to the end of each month, until 31^st^ August 2020. For the second wave, we use data from 1^st^ August 2020, again generating estimates using data available up to the end of each month, until 31^st^ December 2020.

For the first and second waves, the overall length of stay distributions, with uncertainty from parametric bootstrapping with 1,000 repeats, are presented in Table 1. Monthly point estimates of the mean delay, used as input for the short-term forecasts in Section 2.4, are given in Table D.

*SM.1.3 Model fitting procedure*

*SM.1.3.1 Fixed and free parameters*

To reduce the number of free parameters in the model, and therefore to avoid problems with identifiability when fitting the model, we fixed a number of parameters that we were confident in by taking point estimates from the literature and from our estimates of the delay distributions (Tables 2 and A). EpiBeds could be extended to use prior distributions for some or all of these parameters, which may also aid identifiability. The decision to used fixed parameters may reduce some of the uncertainty in the projections, but still resulted in reliable fits and forecasts. The remaining free parameters (Table B) were inferred from the model fitting, using uninformative priors with the exception of *p_D_*, which had a strongly informative prior estimated from the CHESS/SARI data.

**Table A.** *List of fixed parameters for the compartmental model*

| **Variable** | **Description** |
| --- | --- |
| *r_E_* | Rate of transition from early stage infectious classes (E_S_, I_S_, E_A_, I_A_) |
| *r_AR_* | Rate of transition from late stage asymptomatic (A_R_) to recovered (R) |
| *r_LR_* | Rate of transition from late stage symptomatic (L_R_) to recovered (R) |
| *r_LH_* | Rate of transition from late stage severely symptomatic (L_H_) |
| *r_HR_* | Rate of transition from hospital admission (H_R_) to recovery (R), without ICU |
| *r_HC_* | Rate of transition from hospital admission (H_C_) to ICU (C_M_, C_D_) |
| *r_HD_* | Rate of transition from hospital admissions (H_D_) to death (D), without ICU |
| *r_CM_* | Rate of transition from critical care admission (C_M_) to step down (M_R_) |
| *r_CD_* | Rate of transition from critical care admission (C_D_) to death (D) |
| *r_MR_* | Rate of transition from being stepped down (M_R_) to discharge (R) |
| *p_A_* | Proportion of infected individuals that will be asymptomatic |
| *p_H_* | Proportion of symptomatic individuals that will be hospitalised |

**Table B.** *List of free parameters for the compartmental model*

| **Variable** | **Description** |
| --- | --- |
| *I_0_* | Initial number of infected individuals (divided in proportions given by *p_A_* and 1-*p_A_* between the first E_A_ and E_S_ stages) |
| 𝜎_A_ | Overdispersion parameter for admissions data |
| 𝜎_B_ | Overdispersion parameter for beds data |
| 𝜎_C_ | Overdispersion parameter for ICU data |
| 𝜎_D_ | Overdispersion parameter for deaths data |
| *p_C_* | Proportion of hospitalised individuals that will enter critical care |
| *p_T_* | Proportion of hospitalised individuals that will die without entering critical care |
| *p_D_* | Proportion of individuals in critical care that will die (includes a strongly informative prior distribution) |
| 𝛽_1_ | Transmission rate up to 15/03/2020 |
| 𝛽_2_ | Transmission rate from 15/03/2020 to 24/03/2020 |
| 𝛽_3_ | Transmission rate from 24/03/2020 to 11/04/2020 |
| 𝛽_4_ | Transmission rate from 11/04/2020 to 15/08/2020 |
| 𝛽_5_ | Transmission rate from 15/08/2020 to 06/09/2020 |
| 𝛽_6_ | Transmission rate from 06/09/2020 to 14/10/2020 |
| 𝛽_7_ | Transmission rate from 14/10/2020 to 05/11/2020 |
| 𝛽_8_ | Transmission rate from 05/11/2020 to 18/11/2020 |
| 𝛽_9_ | Transmission rate from 18/11/2020 to 10/12/2020 |
| 𝛽_10_ | Transmission rate from 10/12/2020 to 31/12/2020 |

*SM.1.3.2 Prior estimates for outcome probabilities*

To generate prior estimates for the probability of dying on ICU we used the CHESS/SARI dataset, which contains approximately 90% of the English ICU data (based on comparing patient counts to the SITREP data). This suggests SARI ICU outcomes were representative of general ICU outcomes.

Due to the censoring of patient outcomes, and to capture data uncertainty, we estimated outcome probabilities using a non-Markovian competing risk model [8] using MCMC to generate uncertainty [9]. By fitting this model to the data, we can estimate the hazard functions for these competing risks. From these fitted hazard functions, we can simulate posterior distributions for the outcome probabilities, which allows us to quantify uncertainty around the point estimates. We also report the estimates for *p_D_* obtained at the end of each month since 1^st^ March 2020 (Table E), which gives the prior estimates used to generate the short-term forecasting scenarios in Section 2.4.

*SM.1.3.3 Transmission rate changes*

In EpiBeds, the background epidemic is driven by a transmission rate 𝛽, that represents the total infectious pressure exerted by a symptomatic infectious individual. This parameter collates contact behaviour, transmission probability of contacts and strength of contacts into a single parameter. On an individual level, this does not provide accurate information about the transmission dynamics, but on a population level aggregating all of these into a single parameter is a simple way to represent the average transmission dynamics in the population.

To model the background epidemic, we need to estimate the value of this transmission parameter. We cannot assume this is constant, because there are large changes in this parameter as behaviour changes, for example due to lockdown. On the other hand, we do not want to add too many different values, as this risks overfitting noise in the data rather than genuine changes in transmission. There are various clear transmission rates that need to be fitted: the transmission rate that led to the original growth of the outbreak, the transmission rate after lockdown 1, the transmission rate when lockdown 1 is eased, the transmission rate after lockdown 2, and the transmission rate after lockdown 2 is eased. The easing of lockdown 1 was gradual, so it is not easy to specify dates for the transmission rate changes. Through looking at the data on an exponential scale, we notice two changes in the gradient that can be associated with the easing of lockdown 1. Since this data is on hospitalisations, we consider these gradient changes to correspond to transmission changes occurring 9 days prior [5], which corresponds to the median delay between infection and hospital admission. Therefore, we add new transmission rates on 11^th^ April 2020 and 15^th^ August 2020. From the log-scale, we also notice that transmission changed before the first lockdown. This was not sufficient to bring cases down, but slowed down the original rate of growth. Therefore, we add a new transmission rate on 13^th^ March 2020 Transmission trends changed as the second wave became established, so a further change point was added on 6^th^ September 2020. Additionally, although not a national policy, strict restrictions were imposed in the North West in October, before the second national lockdown in November. Since at this time North West had the highest prevalence in England, this led to a substantial change in the national trends. Therefore, we add another transmission change on 14^th^ October 2020 to correspond to the “tier 3” restrictions imposed in Liverpool. Although the second national lockdown was lifted on 2^nd^ December 2020, it became apparent that transmission had already increased before this date, likely driven by a combination of increased movement towards the end of lockdown and the emergence of the more transmissible B.1.1.7 variant [10]. To capture this, investigating the data on a log-scale suggests a transmission change should be added on 18^th^ November 2020. This transmission change also encompasses any transmission change from lifting the second lockdown, so we do not explicitly add the 2^nd^ December 2020 change point.

In theory, interventions can be added to EpiBeds as soon as they have occurred. However, with a median delay of 9 days from infection to hospital admission, there may be insufficient data to inform EpiBeds on the impact of the intervention immediately after their implementation. To remove unnecessary uncertainty, if an intervention occurs within the last 7 days of data, we do not include this, so that the forecast will instead generate a counterfactual where the intervention has no impact on transmission. On the other hand, significant transmission changes that do not correspond to known intervention dates cannot be added until there is sufficient data to observe the change in trend. In practice, we only consider such changes once half a month has passed, by which point there will be sufficient data to quantify the change.

In addition to these fixed transmission changes, when generating forecasts we consider a final change point 3 weeks prior to the final data point, unless there is another change point in this interval. This is to allow EpiBeds to react to changes in transmission to predict a current value of transmission, though this will be a transmission averaged over the last 3 weeks rather than a now-cast. A 3-week window was chosen as a bespoke compromise between an interval that is too long, which would give narrow uncertainty and estimates of *R_e_(t)* excessively slow at responding to visible changes in the data trends, and one that is too short to ensure there is sufficient data to inform the final transmission rate (recall that, with an average delay of 9 days from infection to hospitalisation, the last few data points provide less and less information on transmission as they approach the time at which projections are made) and estimates would be dominated by random noise and hard-to-control discrepancies between the signal from different data streams. When this 3-week transmission change suggests a substantial change to the previous trend, this prompts investigations into whether a new fixed transmission change should be added, following the procedure described above.

Through this, we obtain a system of ordinary differential equations with a time varying parameter beta, which changes values according to a step function. The jump times of this step function are pre-specified, but the resulting values are fitted to the data.

*SM.1.3.4 Evaluating the accuracy of short-term forecasting*

Throughout the pandemic, EpiBeds was continuously refined. Therefore, instead of analysing the historic forecasts that were generated, we generate new forecasts retrospectively, using data that were available at the time. We generated two forecasts per month, one on the first day of each month and one on the 15th day, using SITREP and CPNS data up to the day before the first day of forecasts. We removed the last two days of hospital admission data and the last day of the deaths data since these data points are lagged and will be revised upwards. For the length of stay estimates and prior *p_D_* estimate, we use data up to the end of each month. Therefore, for forecasts generated on the 1st, the data run up to the previous day, whereas for the 15th the time series from the SITREP and CPNS are used until the 14^th^ (minus two days for admissions and five for deaths) while the data for the length of stay and the prior estimate of *p_D_* run up to the end of the previous month. It is possible to continuously generate these estimates in real-time, but this is not reflective of the workflow in practice.

As described in Section 2.3.1, transmission rates change at pre-specified dates in EpiBeds. Some of these correspond to large policy changes, so can be added as soon as we expect them to become visible in the data (7 days after policy change). Others correspond to noticeable changes in the gradient of the admissions data on a log scale. These are only added once there is sufficient data to know that there has been a genuine change in the trend, which generally occurs 15 days after the change point. Finally, when fitting EpiBeds, a final change point is added three weeks prior to the last data point, provided there is no other change point in this window. Therefore, when generating the scenarios presented in Section 2.4, the changes points are specified as indicated in Table C.

**Table C.** *Dates of transmission rate changes for the 19 scenarios considered in Section 2.4. For the 15^th^ April 2020 forecast, a change point is not added three weeks before the last data point since this would be one day after lockdown, and therefore risks overfitting the data.*

| **Forecast start date** | **1** | **2** | **3** | **4** | **5** | **6** | **7** | **8** | **9** |
| --- | --- | --- | --- | --- | --- | --- | --- | --- | --- |
| **01/04/20** | 13/03/20 | 24/03/20 | - | - | - | - | - | - | - |
| **15/04/20** | 13/03/20 | 24/03/20 | - | - | - | - | - | - | - |
| **01/05/20** | 13/03/20 | 24/03/20 | 09/04/20 | - | - | - | - | - | - |
| **15/05/20** | 13/03/20 | 24/03/20 | 11/04/20 | 24/04/20 | - | - | - | - | - |
| **01/06/20** | 13/03/20 | 24/03/20 | 11/04/20 | 10/05/20 | - | - | - | - | - |
| **15/06/20** | 13/03/20 | 24/03/20 | 11/04/20 | 25/05/20 | - | - | - | - | - |
| **01/07/20** | 13/03/20 | 24/03/20 | 11/04/20 | 09/06/20 | - | - | - | - | - |
| **15/07/20** | 13/03/20 | 24/03/20 | 11/04/20 | 24/06/20 | - | - | - | - | - |
| **01/08/20** | 13/03/20 | 24/03/20 | 11/04/20 | 10/07/20 | - | - | - | - | - |
| **15/08/20** | 13/03/20 | 24/03/20 | 11/04/20 | 25/07/20 | - | - | - | - | - |
| **01/09/20** | 13/03/20 | 24/03/20 | 11/04/20 | 10/08/20 | - | - | - | - | - |
| **15/09/20** | 13/03/20 | 24/03/20 | 11/04/20 | 15/08/20 | 25/08/20 | - | - | - | - |
| **01/10/20** | 13/03/20 | 24/03/20 | 11/04/20 | 15/08/20 | 09/09/20 | - | - | - | - |
| **15/10/20** | 13/03/20 | 24/03/20 | 11/04/20 | 15/08/20 | 06/09/20 | 24/09/20 | - | - | - |
| **01/11/20** | 13/03/20 | 24/03/20 | 11/04/20 | 15/08/20 | 06/09/20 | 14/10/20 | - | - | - |
| **15/11/20** | 13/03/20 | 24/03/20 | 11/04/20 | 15/08/20 | 06/09/20 | 14/10/20 | 05/11/20 | - | - |
| **01/12/20** | 13/03/20 | 24/03/20 | 11/04/20 | 15/08/20 | 06/09/20 | 14/10/20 | 05/11/20 | 09/11/20 | - |
| **15/12/20** | 13/03/20 | 24/03/20 | 11/04/20 | 15/08/20 | 06/09/20 | 14/10/20 | 05/11/20 | 18/11/20 | 24/11/20 |
| **01/01/21** | 13/03/20 | 24/03/20 | 11/04/20 | 15/08/20 | 06/09/20 | 14/10/20 | 05/11/20 | 18/11/20 | 10/12/20 |

**SM.2 Supplementary Figures**

*.*
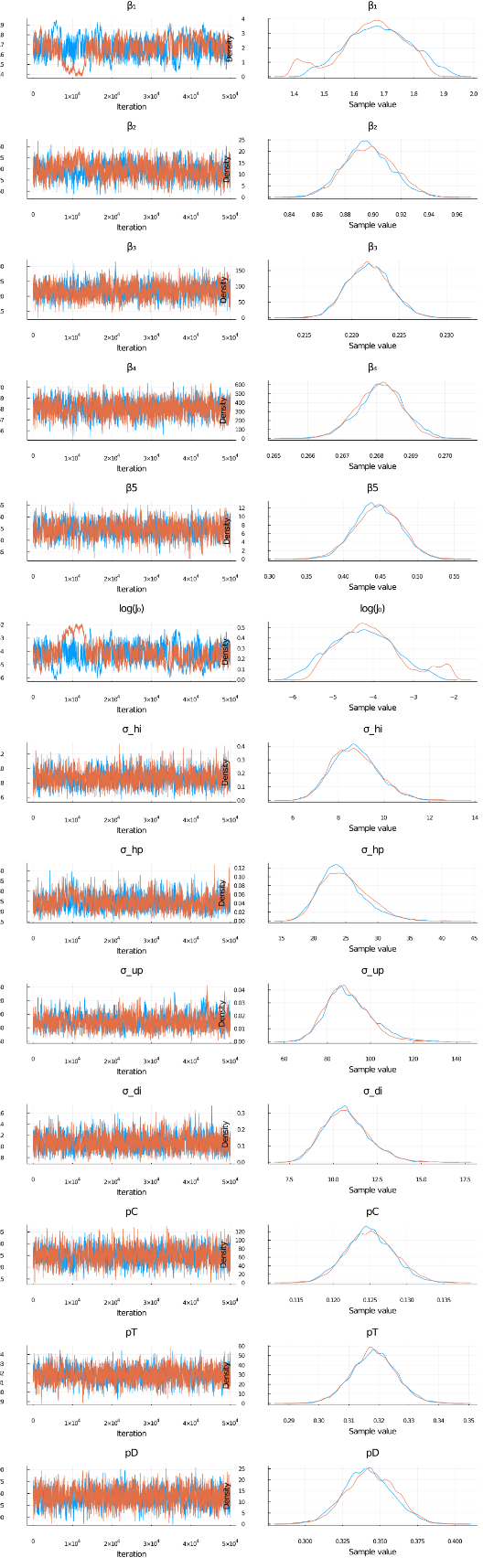
*
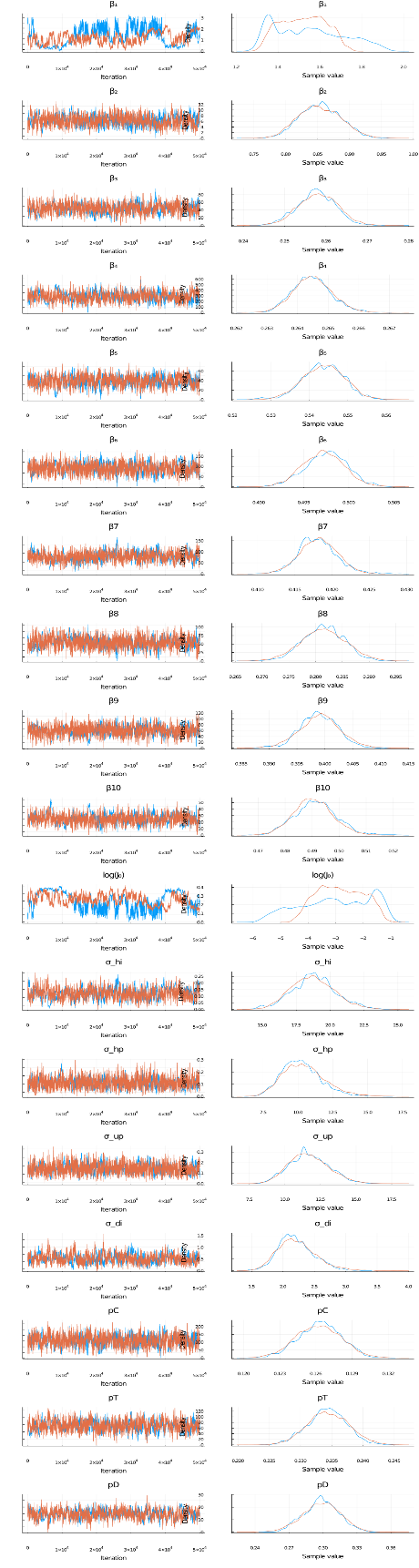
*

**Fig A.** *MCMC trace plots for EpiBeds for the results shown in Figs 2 and 3. Note that β_1_ and J_0_ are highly correlated and poorly identified.*


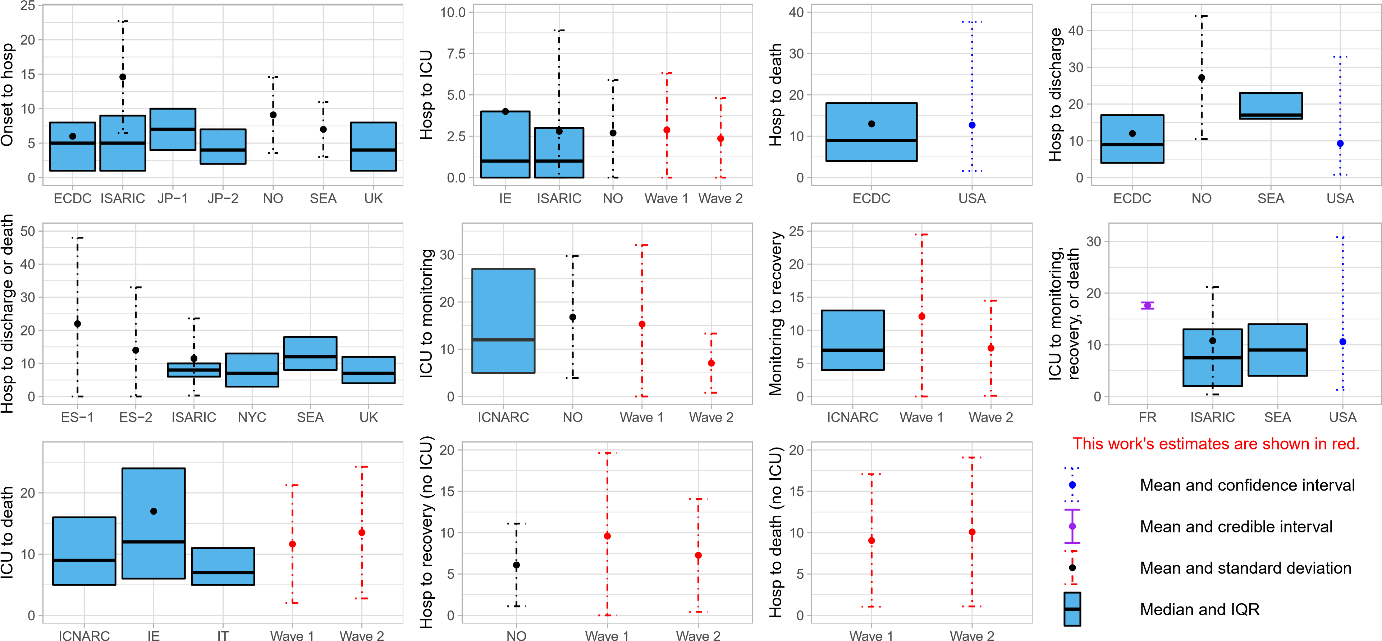


**Fig B. *Length of stay and delay distributions relating to hospitalisations in Europe and North America.*** *Estimates of lengths of delays prior to hospital admissions, and the lengths of stay through hospital patient pathways from a variety of studies. The estimates calculated here are shown in red for wave 1 (01/03/2020 - 31/08/2020) and wave 2 (01/08/2020 - 31/12/2020). Other estimates for comparison are: aggregated values from the European Centre for Disease Prevention and Control (ECDC,* [12]*); ISARIC reports (ISARIC,* [13]*); the Norwegian Institute of Public Health (NO,* [14]*); a study among Seattle patients (SEA,* [15]*), ISARIC patients in the UK (UK,* [16]*); a study of severely ill patients in New York (NYC,* [17]*); Irish reported data to the ECDC (IE,* [12]*); ICNARC reports (ICNARC,* [18]*); a study on ICU patients in Lombardy (IT,* [19]*); a cohort study in California and Washington (USA,* [20]*); Japanese hospital patients (JP,* [21]*); Spanish hospital patients in Reus (ES,* [22]*); and French hospitalisation data (FR,* [23]*). All estimates are from the first wave (although the dates may not correspond to the dates used here), except for JP and ES, for which data from the first two waves are given. Mean values are marked by dots, and median values and interquartile ranges by box plots. All error bars denote standard deviations (dot-dashed), except for FR where these bounds denote the 95% credible interval (solid), and USA where the bounds denote the 95% confidence interval (dotted), and therefore cannot be directly compared. There is a marked skew in several distributions as seen by the difference between mean and median values. We do not include estimates from China due to documented evidence that these distributions differ* [24,25]*.*


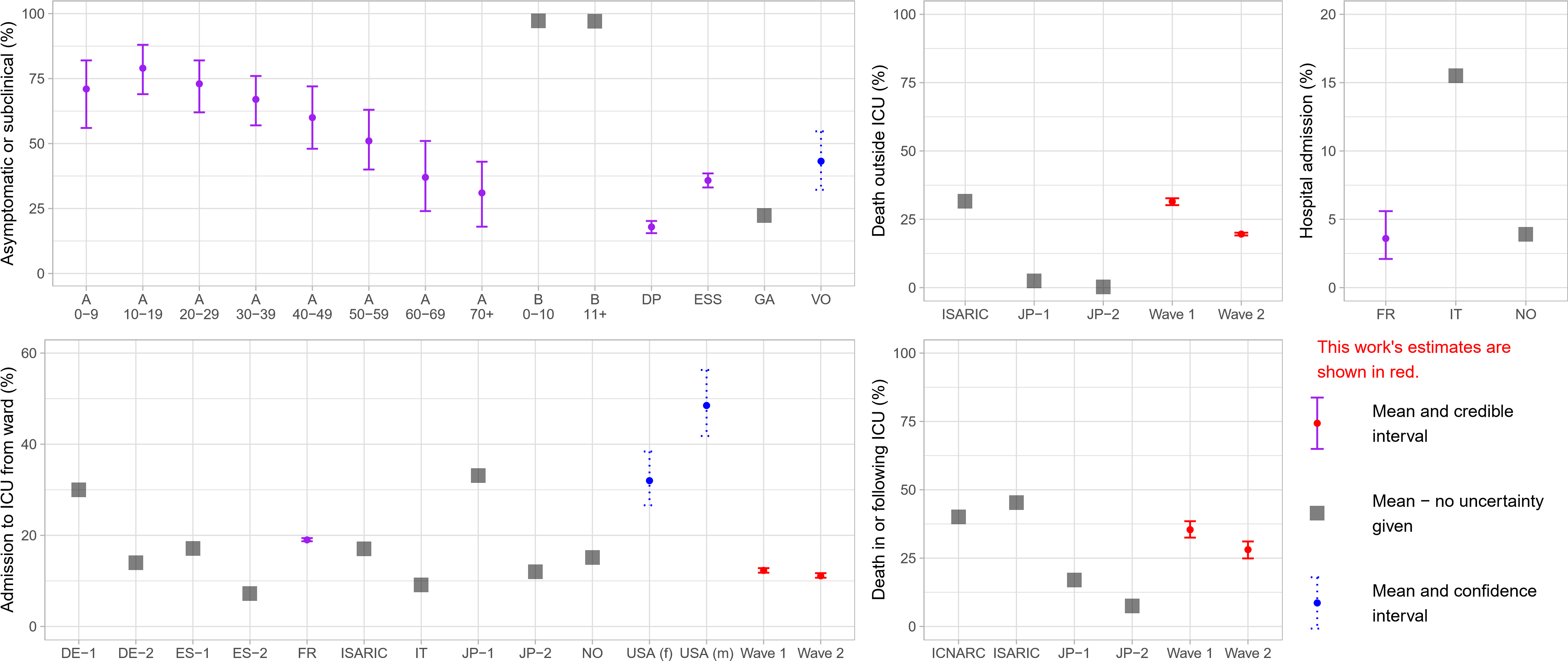


**Fig C. *Probabilities of asymptomatic individuals in the general population and different outcomes through the hospital pathway.*** *We present our own estimates of the probability of different hospital outcomes (red, labelled as Wave 1 and Wave 2) compared to other estimates from the literature. In addition, we include estimates from the literature of the proportion of individuals with asymptomatic infection and the percentage of individuals admitted to hospital. The included studies from the literature include estimates broken down across multiple age groups (A,* [26]*), estimates for Chinese children (B,* [27]*), population averages in the German town of Gangelt (GA,* [28]*), on the Diamond Princess cruise ship (DP,* [29]*), across a Spanish serological survey (ESS,* [30]*), in a cohort study in California and Washington (USA,* [20]*) broken down by sex (m, f), and in the Italian municipality of Vo' (VO,* [31]*). Estimates are also obtained from ISARIC (ISARIC,* [13]*), ICNARC (ICNARC,* [18]*), France (FR,* [23]*), Italy (IT,* [32]*), Japanese hospital patients (JP,* [21]*), Spanish hospital patients in Reus (ES,* [22]*), German hospitalisations (DE,* [33]*), and Norway (NO,* [14]*). If not specified, all estimates are from the first wave. Otherwise, estimates are labelled for each wave but may not agree on the respective date ranges. The proportion of asymptomatic cases varies across studies and by age, whereas the proportion of individuals entering different hospital pathways is fairly consistent. Some of the presented quantities are obtained from estimating the proportions of patients with each outcome, and so no uncertainty is given. In other cases, these probabilities are inferred numerically, and so credible intervals are provided. There is clear geographic variability in hospital and ICU admission, which is known to correlate with age* [32] *and sex.*

**SM.3 Supplemental Results**

**Input parameters for short-term forecasting evaluation**

When generating the short-term forecasts in Section 2.4, we required point estimates for the length of stay and priors for the probability of death on ICU, using data available at the time of each forecast. We generated estimates using data available at the end of each month, from the end of March 2020 onwards, which were used for any forecasts generated in the subsequent month. The resultant estimates are given in Tables D and E.

In Table D, we present the monthly length of stay estimates, which represent the best estimates available up to the end of the given month. Looking at the estimates over time, we observe that although the method (Section SM.1.2) attempts to compensate for the truncated tail observations, it can take a while for it to receive enough information to provide reliable estimates: moving from March to May, the mean lengths of stay gradually increase, as more information arrives with which to parameterise the tail of the distribution. This is to be expected, since in March and April, there was a maximum of 31 and 61 days, respectively, for the observed lengths of stay, so any tail observations longer than this would not be identified, and therefore EpiBeds does not have sufficient information to correctly adjust for the truncated tail. From May to August, the estimates are mostly stable. A similar pattern occurs as we move from September to December, whereby it takes a couple of months for the estimates to stabilise.

In Table E, we present the monthly prior estimates for *p_D_*. Initial *p_D_* estimates were very high, likely driven by patients that recover being more affected by right-censoring in their outcomes, since the length of stay to discharge is longer than the length of stay to death. In the second wave, initial *p_D_* estimates were low. At this point, the length of stay to death was longer than to discharge, so here the deaths are more affected by the right truncation. Additionally, during the second wave the age distribution of admissions changed as the epidemic progressed, resulting in older patients being admitted, who had a higher rate of mortality in ICU.

**Table D.** *Point estimates for the mean length of stay using data until the end of each month. From March to August, this uses all data from 1^st^ March 2020. From September to December, this uses all data from 1^st^ August 2020.*

| **Length of stay** | **March** | **April** | **May** | **June** | **July** | **August** | **September** | **October** | **November** | **December** |
| --- | --- | --- | --- | --- | --- | --- | --- | --- | --- | --- |
| **Hosp to ICU** | 1.46 | 2.1 | 2.49 | 2.65 | 2.86 | 2.79 | 1.82 | 2.62 | 2.53 | 2.70 |
| **ICU to death** | 7.61 | 9.96 | 11.10 | 11.4 | 11.55 | 11.84 | 9.17* | 14.71 | 14.88 | 15.33 |
| **ICU to monitoring** | 5.58 | 11.25 | 16.5 | 16.63 | 16.10 | 15.93 | 6.51 | 6.86 | 7.46 | 8.57 |
| **Monitoring to recovery** | 6.91 | 6.72 | 9.29 | 10.76 | 11.31 | 11.85 | 4.32 | 6.59 | 6.27 | 6.45 |
| **Hosp to recovery (no ICU)** | 5.13 | 8.19 | 8.91 | 9.20 | 9.24 | 9.37 | 6.89 | 10.04 | 10.07 | 10.02 |
| **Hosp to death (no ICU** | 8.09 | 7.79 | 8.45 | 8.68 | 8.86 | 8.93 | 5.20 | 10.46* | 13.37 | 12.16 |

**Table E.** *Prior estimates for the probability of dying on ICU, using data until the end of each month. From March to August, all data from 1^st^ March 2020 is used. From September to December, all data from 1^st^ August 2020 is used.*

| ***p_D_*** | **March** | **April** | **May** | **June** | **July** | **August** | **September** | **October** | **November** | **December** |
| --- | --- | --- | --- | --- | --- | --- | --- | --- | --- | --- |
| **mean** | 0.46 | 0.42 | 0.372 | 0.355 | 0.358 | 0.357 | 0.159 | 0.252 | 0.293 | 0.305 |
| **standard deviation** | 0.0335 | 0.018 | 0.0215 | 0.018 | 0.018 | 0.017 | 0.033 | 0.026 | 0.0195 | 0.0165 |

**Supplementary material – references**

1. Deaths caused by COVID-19 only and the difference between ONS and PHE figures - Office for National Statistics [Internet]. [cited 2021 Sep 16]. Available from: https://www.ons.gov.uk/aboutus/transparencyandgovernance/freedomofinformationfoi/deathscausedbycovid19onlyandthedifferencebetweenonsandphefigures

2. UK Gov COVID dashboard [Internet]. Available from: https://coronavirus.data.gov.uk/details/download

3. Sun J. Empirical Estimation of a Distribution Function with Truncated and Doubly Interval-Censored Data and Its Application to AIDS Studies. Biometrics. 1995;51(3):1096–104.

4. Overton CE, Stage HB, Ahmad S, Curran-Sebastian J, Dark P, Das R, et al. Using statistics and mathematical modelling to understand infectious disease outbreaks: COVID-19 as an example. Infectious Disease Modelling. 2020 Jan 1;5:409–41.

5. Pellis L, Scarabel F, Stage HB, Overton CE, Chappell LHK, Fearon E, et al. Challenges in control of COVID-19: short doubling time and long delay to effect of interventions. Philosophical Transactions of the Royal Society B: Biological Sciences. 2021 Jul 19;376(1829):20200264.

6. Cox DR. Cox, D. R. (1962). Renewal Theory, Methuen and Co. Ltd., London. 1962.

7. Vogel T. Théorie des systèmes évolutifs. Vol. 22. Gauthier-Villars; 1965.

8. Vekaria B, Overton C, Wiśniowski A, Ahmad S, Aparicio-Castro A, Curran-Sebastian J, et al. Hospital length of stay for COVID-19 patients: Data-driven methods for forward planning. BMC Infectious Diseases. 2021 Jul 22;21(1):700.

9. Overton CE. MultiStateLOS [Internet]. Available from: https://github.com/thomasallanhouse/covid19-los/tree/master/forecasting_model_and_tutorial

10. Davies NG, Abbott S, Barnard RC, Jarvis CI, Kucharski AJ, Munday JD, et al. Estimated transmissibility and impact of SARS-CoV-2 lineage B.1.1.7 in England. Science. 2021 Apr 9;372(6538):eabg3055.

11. Overton CE. EpiBeds [Internet]. Available from: https://github.com/OvertonC/EpiBeds

12. ECDC. COVID-19 surveillance report Week 25, 2020 [Internet]. 2020 [cited 2021 Sep 10]. Available from: https://covid19-surveillance-report.ecdc.europa.eu/

13. ISARIC. ISARIC COVID-19 Report: 08 June 2020 [Internet]. 2020 [cited 2021 Sep 10]. Available from: https://isaric.tghn.org/

14. FHI COVID-19 modelling team. Coronavirus modelling at the NIPH. Retrieved June 12, 2020. [Internet]. Norwegian Institute of Public Health. 2020 [cited 2021 Sep 10]. Available from: https://www.fhi.no/en/id/infectious-diseases/coronavirus/coronavirus-modelling-at-the-niph-fhi/

15. Bhatraju PK, Ghassemieh BJ, Nichols M, Kim R, Jerome KR, Nalla AK, et al. Covid-19 in Critically Ill Patients in the Seattle Region — Case Series. New England Journal of Medicine. 2020 May 21;382(21):2012–22.

16. Docherty AB, Harrison EM, Green CA, Hardwick H, Pius R, Norman L, et al. Features of 16,749 hospitalised UK patients with COVID-19 using the ISARIC WHO Clinical Characterisation Protocol [Internet]. 2020 Apr [cited 2021 Sep 10] p. 2020.04.23.20076042. Available from: https://www.medrxiv.org/content/10.1101/2020.04.23.20076042v1

17. Petrilli CM, Jones SA, Yang J, Rajagopalan H, O’Donnell L, Chernyak Y, et al. Factors associated with hospital admission and critical illness among 5279 people with coronavirus disease 2019 in New York City: prospective cohort study. BMJ. 2020 May 22;369:m1966.

18. ICNARC. ICNARC report on COVID-19 in critical care 17 July 2020 [Internet]. 2020 [cited 2021 Sep 10]. Available from: https://www.icnarc.org/Our-Audit/Audits/Cmp/Reports

19. Grasselli G, Zangrillo A, Zanella A, Antonelli M, Cabrini L, Castelli A, et al. Baseline characteristics and outcomes of 1591 patients infected with SARS-CoV-2 admitted to ICUs of the Lombardy Region, Italy. Jama. 2020;323(16):1574–81.

20. Lewnard JA, Liu VX, Jackson ML, Schmidt MA, Jewell BL, Flores JP, et al. Incidence, clinical outcomes, and transmission dynamics of severe coronavirus disease 2019 in California and Washington: prospective cohort study. BMJ. 2020 May 22;369:m1923.

21. Saito S, Asai Y, Matsunaga N, Hayakawa K, Terada M, Ohtsu H, et al. First and second COVID-19 waves in Japan: A comparison of disease severity and characteristics. Journal of Infection. 2021 Apr 1;82(4):84–123.

22. Iftimie S, López-Azcona AF, Vallverdú I, Hernández-Flix S, De Febrer G, Parra S, et al. First and second waves of coronavirus disease-19: A comparative study in hospitalized patients in Reus, Spain. PloS one. 2021;16(3):e0248029.

23. Salje H, Tran Kiem C, Lefrancq N, Courtejoie N, Bosetti P, Paireau J, et al. Estimating the burden of SARS-CoV-2 in France. Science. 2020 Jul 10;369(6500):208–11.

24. Rees EM, Nightingale ES, Jafari Y, Waterlow NR, Clifford S, Pearson CA, et al. COVID-19 length of hospital stay: a systematic review and data synthesis. BMC medicine. 2020;18(1):1–22.

25. Sanche S, Lin YT, Xu C, Romero-Severson E, Hengartner N, Ke R. High Contagiousness and Rapid Spread of Severe Acute Respiratory Syndrome Coronavirus 2. Emerg Infect Dis. 2020 Jul;26(7):1470–7.

26. Davies NG, Klepac P, Liu Y, Prem K, Jit M, Eggo RM. Age-dependent effects in the transmission and control of COVID-19 epidemics. Nat Med. 2020 Aug;26(8):1205–11.

27. Dong Y, Mo X, Hu Y, Qi X, Jiang F, Jiang Z, et al. Epidemiological characteristics of 2143 pediatric patients with 2019 coronavirus disease in China. Pediatrics. 2020;

28. Streeck H, Schulte B, Kümmerer BM, Richter E, Höller T, Fuhrmann C, et al. Infection fatality rate of SARS-CoV-2 infection in a German community with a super-spreading event [Internet]. 2020 Jun [cited 2021 Sep 10] p. 2020.05.04.20090076. Available from: https://www.medrxiv.org/content/10.1101/2020.05.04.20090076v2

29. Mizumoto K, Kagaya K, Zarebski A, Chowell G. Estimating the asymptomatic proportion of coronavirus disease 2019 (COVID-19) cases on board the Diamond Princess cruise ship, Yokohama, Japan, 2020. Eurosurveillance. 2020 Mar 12;25(10):2000180.

30. Pollán M, Pérez-Gómez B, Pastor-Barriuso R, Oteo J, Hernán MA, Pérez-Olmeda M, et al. Prevalence of SARS-CoV-2 in Spain (ENE-COVID): a nationwide, population-based seroepidemiological study. The Lancet. 2020 Aug 22;396(10250):535–44.

31. Lavezzo E, Franchin E, Ciavarella C, Cuomo-Dannenburg G, Barzon L, Del Vecchio C, et al. Suppression of a SARS-CoV-2 outbreak in the Italian municipality of Vo’. Nature. 2020 Aug;584(7821):425–9.

32. Riccardo F, Ajelli M, Andrianou XD, Bella A, Manso MD, Fabiani M, et al. Epidemiological characteristics of COVID-19 cases and estimates of the reproductive numbers 1 month into the epidemic, Italy, 28 January to 31 March 2020. Eurosurveillance. 2020 Dec 10;25(49):2000790.

33. Karagiannidis C, Windisch W, McAuley DF, Welte T, Busse R. Major differences in ICU admissions during the first and second COVID-19 wave in Germany. The Lancet Respiratory Medicine. 2021 May 1;9(5):e47–8.
