# Supplementary material for: EpiBeds: Data informed modelling of the COVID-19 hospital burden in England
Source: PLoS Comput Biol. 2022 Sep 6;18(9):e1010406. doi: 10.1371/journal.pcbi.1010406 (PMC9481171; doi:10.1371/journal.pcbi.1010406)
Supplement: Attachment — Submitted filename: EpiBeds review.docx [file pcbi.1010406.s002.docx]

In this paper, the authors describe their mathematical model EpiBeds, which has been developed and used to forecast hospital bed occupancy by patients with COVID-19 in the United Kingdom. Alongside a detailed explanation of the model structure and the different data sources that were used to build and parameterise it, they also discuss its short term forecasting performance. I enjoyed learning about this work, and think it will be relevant to readers of PLOS Computational Biology, as it is highly technical and presents a useful framework for hospital bed occupancy forecasting.

My only major worry is the length of the manuscript. Although the text is clear, with sufficient details to be understood, there are a few instances where information is unnecessarily repeated, and some sections of the manuscript could be more concise. I know this is subjective, but I think this may discourage some readers from reading this article, which would be a shame since the work it describes is excellent. I also have some minor comments that I hope the authors will find useful and take into consideration to further improve their manuscript. I have listed these comments addressed to the authors below.

**Introduction**

Parts of the Introduction after the first two paragraphs read a lot like a Discussion (particularly the last paragraph), and describe the Results with too much details (eg the paragraph on Re and Rc). I think you could rewrite this section to be much more concise, as readers will essentially find all of this information in the next section. Perhaps instead include here a short presentation of existing bed occupancy forecasting tools, and justification for why you decided to build EpiBeds in this way (How are hospitalisations typically modelled? Why is it important to consider patient pathways and not just average length of stay by ward/ICU bed?).

**Results**

This first paragraph could be removed – there is no need to introduce the model, data streams, short-term forecasts and Re here. I understand that this is a summary paragraph, but these elements come much later in the Results, so you could just introduce them immediately before going into details. In any case, this brief summary of your results will already be present in the Introduction, so readers will already be aware of where this is going.

Figure 1: could you make the keys in the legend slightly bigger/longer, like in Figure 6? This would make it easier to distinguish between the dotted and dot-dashed lines. It would also be helpful to make it clearer in the legend that the red lines are your estimates, while the others are all data (I know this is in the caption, but at some readers may be confused and treat the red lines as aggregated data values for waves 1 and 2).

Section 2.1: “these pathways have a relatively minor contribution…” could you please provide numbers to support this claim? Perhaps number of occurrences of these minor pathways in the dataset versus occurrences of the pathways you kept?

Section 2.1 and throughout (e.g. 1^st^ sentence of 2.3.2): I was slightly confused regarding the dates for the waves, and the dates for the data you used to fit each wave. Perhaps a sentence here clearly stating the beginning and end dates for each wave, and the beginning and end dates for the data used to fit the model for each wave, would be useful to indicate that these are not the same. Reading this the first time, I assumed that there was a mistake and that 01/08/2020 should be 01/09/2020.

Figure 3: in the text you add numerical subscripts to repeated compartments (LH and E), but these are absent from the figure and should be added there too.

Section 2.3.1: typo, space missing in sentence “(Section M.4.3)for wave”

Figures 4 & 5: could you please clarify how you calculated the 95% prediction interval?

Section 2.3.3: “Using uninformative priors, EpiBeds did not provide reliable estimates for these three probabilities, though it still fitted the data well”. You then explain that you used an informative prior for pd, but were unable to do so for pc and pt due to lack of data. Does that mean that the estimates in Table 3 are still unreliable, since they still rely on uninformative priors? Yet as you point out, they are roughly similar to other available estimates (Figure 6) …

Section 2.3.3: consider rephrasing “though a significant reduction is clearly visible” to “though a significant reduction between waves one and two is clearly visible” for improved clarity

Table 3: to help make this table standalone, please add a column to state what pd, pc, pt correspond to, or at least add this information to the caption.

Figure 6: what is the source for the estimates by age groups in the top left? I assume these were not generated by your model, as it is not stratified by age?

Figure 6: could you please adjust the x-axis labels to improve readability? It is particularly hard to distinguish between ICNARC/ISARIC (top right) and the age groups (top left).

Section 2.3.4 & Figure 4: I think the 15^th^ August transmission rate change is not present in the Figure? I can only see one line for August there.

Table 4: please clarify what you mean by “very few data points” – less than what threshold?

**Methods**

Discussion/Section M.3: an implicit assumption of your model is that recovery or death are final endpoints, and that it is not possible for recovered individuals to become susceptible again. I agree this is a reasonable assumption for the period you focused on (March-December 2020), but it should still be flagged as a limitation of the model in the Discussion, as it would make the model unreliable over longer durations (due to waning immunity, or variants escaping existing immunity as we are currently seeing).

Section M.4: you could remove the first paragraph to be more concise.

Section M.4.1: I may be missing something, but why is pd not a fixed parameter? According to M.4.3, CHESS/SARI contains ~90% of English ICU data, which should be sufficient to obtain a robust estimate for pd, instead of just using this data to formulate a prior distribution? In addition, in section 2.3.3 you state that the posterior for pd is very similar to the prior anyway.

Section M.4.6: this section is essentially fully repeated in the Results section, so it may be possible to remove this section or the corresponding section in Results.
