# Supplementary material for: EpiBeds: Data informed modelling of the COVID-19 hospital burden in England
Source: PLoS Comput Biol. 2022 Sep 6;18(9):e1010406. doi: 10.1371/journal.pcbi.1010406 (PMC9481171; doi:10.1371/journal.pcbi.1010406)
Supplement: Attachment — Submitted filename: Epibeds response to reviewers.docx [file pcbi.1010406.s003.docx]

*-Thank you very much for providing these comments on our manuscript; we appreciate the time and effort of the reviewers. The most substantive comments related to the presentation of the model and results, rather than problems with the modelling itself. We have therefore made a substantial effort to restructure the paper, and clarify points where necessary. The paper is now considerably more condensed and much easier to follow. We thank the reviewers for prompting us to do this and highlighting where our explanations were less than clear. Please find below our responses to all of the reviewers comments. The changes include moving most of the Methods to Supplementary Material.*

**Reviewer 1**

In this paper, the authors describe their mathematical model EpiBeds, which has been developed and used to forecast hospital bed occupancy by patients with COVID-19 in the United Kingdom. Alongside a detailed explanation of the model structure and the different data sources that were used to build and parameterise it, they also discuss its short term forecasting performance. I enjoyed learning about this work, and think it will be relevant to readers of PLOS Computational Biology, as it is highly technical and presents a useful framework for hospital bed occupancy forecasting.

*-Thank you very much for these positive comments.*

My only major worry is the length of the manuscript. Although the text is clear, with sufficient details to be understood, there are a few instances where information is unnecessarily repeated, and some sections of the manuscript could be more concise. I know this is subjective, but I think this may discourage some readers from reading this article, which would be a shame since the work it describes is excellent. I also have some minor comments that I hope the authors will find useful and take into consideration to further improve their manuscript. I have listed these comments addressed to the authors below.

*-On coming back to our manuscript after a break, we agree with the reviewer. We have made an effort to make the paper more concise and less repetitive throughout. Thank you for your helpful comments below.*

**Introduction**

Parts of the Introduction after the first two paragraphs read a lot like a Discussion (particularly the last paragraph), and describe the Results with too much details (eg the paragraph on Re and Rc). I think you could rewrite this section to be much more concise, as readers will essentially find all of this information in the next section. Perhaps instead include here a short presentation of existing bed occupancy forecasting tools, and justification for why you decided to build EpiBeds in this way (How are hospitalisations typically modelled? Why is it important to consider patient pathways and not just average length of stay by ward/ICU bed?).

*-We have streamlined the Introduction as suggested.*

**Results**

This first paragraph could be removed – there is no need to introduce the model, data streams, short-term forecasts and Re here. I understand that this is a summary paragraph, but these elements come much later in the Results, so you could just introduce them immediately before going into details. In any case, this brief summary of your results will already be present in the Introduction, so readers will already be aware of where this is going.

*- In light of the referees’ comments the paper has now been significantly restructured to avoid repetition.*

Figure 1: could you make the keys in the legend slightly bigger/longer, like in Figure 6? This would make it easier to distinguish between the dotted and dot-dashed lines. It would also be helpful to make it clearer in the legend that the red lines are your estimates, while the others are all data (I know this is in the caption, but at some readers may be confused and treat the red lines as aggregated data values for waves 1 and 2).

*-We have changed the legend as suggested*. *These figures have now also been moved to the supplemental information as they were tangential to the main focus of the paper.*

Section 2.1: “these pathways have a relatively minor contribution…” could you please provide numbers to support this claim? Perhaps number of occurrences of these minor pathways in the dataset versus occurrences of the pathways you kept?

*-This statement has now been removed*.

Section 2.1 and throughout (e.g. 1^st^ sentence of 2.3.2): I was slightly confused regarding the dates for the waves, and the dates for the data you used to fit each wave. Perhaps a sentence here clearly stating the beginning and end dates for each wave, and the beginning and end dates for the data used to fit the model for each wave, would be useful to indicate that these are not the same. Reading this the first time, I assumed that there was a mistake and that 01/08/2020 should be 01/09/2020.

*-We have clarified the dates of the waves, and the dates used for the model fitting and model projections throughout.*

Figure 3: in the text you add numerical subscripts to repeated compartments (LH and E), but these are absent from the figure and should be added there too.

*-This has now been fixed.*

Section 2.3.1: typo, space missing in sentence “(Section M.4.3)for wave”

*-Corrected*

Figures 4 & 5: could you please clarify how you calculated the 95% prediction interval?

*-This has been added.*

Section 2.3.3: “Using uninformative priors, EpiBeds did not provide reliable estimates for these three probabilities, though it still fitted the data well”. You then explain that you used an informative prior for pd, but were unable to do so for pc and pt due to lack of data. Does that mean that the estimates in Table 3 are still unreliable, since they still rely on uninformative priors? Yet as you point out, they are roughly similar to other available estimates (Figure 6) …

*-We now explain this more clearly in the main text: “For the remaining parameters (Table 2b) we used uninformative priors with the exception of the probabilities of death if in ICU (p_D_). This is because the data on deaths and recoveries do not distinguish whether individuals have transitioned to ICU or not, and hence are both affected simultaneously by a combination of p_C_ and p_D_ (through ICU) and p_T_ (without passing through ICU) thus making these three parameters only weakly identifiable (at best) if at least one of them is not constrained separately. For p_D_ we used a strongly informative Normal prior distribution with a mean and 95% CI estimated from CHESS/SARI data for wave one at 35.7% (31.9%, 38.4%) and for wave 2 at 28.7% (26.5%, 32.1%)”*

Section 2.3.3: consider rephrasing “though a significant reduction is clearly visible” to “though a significant reduction between waves one and two is clearly visible” for improved clarity

*-This has now been rephrased.*

Table 3: to help make this table standalone, please add a column to state what pd, pc, pt correspond to, or at least add this information to the caption.

*-We’ve added the information to the table.*

Figure 6: what is the source for the estimates by age groups in the top left? I assume these were not generated by your model, as it is not stratified by age?

*-These are results from a different study, which reported by age. As in figure 1, we now state in the legend (as well as the caption) that only the estimates in red come from this study. In addition, we have moved both figures to the supplemental material, as they are tangential to the main message of the paper.*

Figure 6: could you please adjust the x-axis labels to improve readability? It is particularly hard to distinguish between ICNARC/ISARIC (top right) and the age groups (top left).

*-Done.*

Section 2.3.4 & Figure 4: I think the 15^th^ August transmission rate change is not present in the Figure? I can only see one line for August there.

*-Corrected.*

Table 4: please clarify what you mean by “very few data points” – less than what threshold?

*-There is no threshold, it can be seen from the trace plots that these data points are not informative as the model struggles to capture both B0 and J0 well, since these are coupled initial conditions. We now explain this more fully.*

**Methods**

Discussion/Section M.3: an implicit assumption of your model is that recovery or death are final endpoints, and that it is not possible for recovered individuals to become susceptible again. I agree this is a reasonable assumption for the period you focused on (March-December 2020), but it should still be flagged as a limitation of the model in the Discussion, as it would make the model unreliable over longer durations (due to waning immunity, or variants escaping existing immunity as we are currently seeing).

*-We have added a paragraph to the Discussion discussing this limitation and noting that vaccination/waning can be added if needed.*

Section M.4: you could remove the first paragraph to be more concise.

*-We have edited the paper throughout for conciseness and to avoid repetition.*

Section M.4.1: I may be missing something, but why is pd not a fixed parameter? According to M.4.3, CHESS/SARI contains ~90% of English ICU data, which should be sufficient to obtain a robust estimate for pd, instead of just using this data to formulate a prior distribution? In addition, in section 2.3.3 you state that the posterior for pd is very similar to the prior anyway.

*-There is still a lot of uncertainty in the estimates for these, and we found that the added flexibility of the prior improved the model fitting.*

Section M.4.6: this section is essentially fully repeated in the Results section, so it may be possible to remove this section or the corresponding section in Results.

*-We have restructured the paper to avoid repetition. We have also opted to have a full description of the methods in supplementary material rather than having a separate methods section in the main text. We believe this improves readability and avoids repetition.*

**Reviewer #2: Review of the manuscript 'EpiBeds: Data informed modelling of the COVID-19 hospital burden in England' by Christopher Overton and colleagues, submitted to PLOS Computational Biology.**

Summary

In this manuscript, Overton and colleagues fit a transmission model for SARS-CoV-2 in England together with a hospital progression model to national-level data on hospital admissions, hospital occupancy (including ICUs), length of stay distributions in the hospital and in the intensive care, and hospital discharge and death data. Main goal is to obtain estimates of the durations in the various compartments in hospitals in England. The authors argue that the main value of the model is that has provided weekly forecasts of bed occupancy and admissions during the early stage of SARS-CoV-2 pandemic, and in addition suggest that the model is easily be adapted to apply to different pathogens and countries.

Evaluation

Overall, the methods are laid out clearly, the model code is publicly available, and I have no doubt that the analyses are sound. Also, I believe that the analyses may have helped the English government and public health bodies to anticipate hospital demand.

*-Thank you. Yes, the reviewer is correct that this model has (and continues to be) used to predict hospital usage.*

I do have some reservations as to what the scientific novelty is of the analyses presented in the manuscript is. It is true that the within-hospital progression model is somewhat more complex than most (perhaps all) other transmission models that have been fitted to hospital data, but many aspects that could have made this manuscript stand out are missing.

*-The novelty of this paper largely lies in the formulation of the model itself - the model was designed around the available data, rather than compromising the information in the data to fit a more standard model. This led, for example, to compartments being defined by what happens next (e.g. critical patients are divided according to whether they will eventually recover or die).*

For instance, (1) all analyses are performed for national level data and as far as I can see no attempt has been made to include analyses at the regional of hospital level. This is unfortunate as the national-level data are the resultant of the superposition of local epidemics.

*-The model has been extensively used at sub-national level, which we have now highlighted in the manuscript. The purpose of the paper is to describe the design of model, its parameterisation, and to give an illustration of its utility. Fundamentally, an epidemic at any scale is essentially a superposition of epidemics at smaller scales, and decisions on the right geographical scale to apply models like ours to are always a judgement call: for example, throughout 2020 the UK Govt published estimates of reproduction numbers and projections for the entirety of the UK, but after the 2021 lockdown a decision was made to stop doing so, owing to the differences in dynamics and policies between England, Scotland, Wales and Northern Ireland. Similarly, although projections for England have always been produced, the arrival of the Alpha variant was much more visible in the hospital admissions and bed occupancy in the South Eastern compared to the Northern regions of England. Finally, at times the epidemic dynamics in certain regions were recognised to be influenced by some Trusts more than others, although it could be argued that at the scale of hospitals or Trusts a stochastic model might be more appropriate than the deterministic one presented here.*

(2) As far as I can see no attempt has been made to analyze and fit age-stratified models to age-stratified data, even though it is known that hospitalization rates are strongly age-dependent while age-stratified incidence has also changed quite a bit during the pandemic. This problem is now partially solved by defining different periods for the analyses, but it would have been nice if everything would have been fitted in one go with an age-stratified transmission model.

*-The reviewer is of course correct that age effects are important, as are other effects such as socio-economic status, prior immune status, treatment options, contact rates, and variant. We did not fit age stratified data, because sufficiently detailed age stratified data was not available at the start of the pandemic to properly parameterise the model. Age-stratified data then became available, but only at very coarse age bands (e.g. 0-17, 18-64 and 65+) and fitting them would have required making a large number of assumptions on the mixing and on how the mixing is affected by different control policies (e.g. school closure). We consciously chose to keep the model as simple and data-informed as possible, thus avoiding relying on assumed parameters and mixing matrix structures. In fact, this level of transparency has also turned out to be one of the model’s strongest points: first of all, we were ourselves surprised about the quality of the fitting to the data in the absence of age-stratification; second, the few times when the model forecasts turned out not to be particularly accurate we were able to hypothesise potential explanations, like shifts in the age distribution of cases, that were often going unnoticed in more complex but less transparent models.*

(3) Hardly any formal (in the statistical sense) effort been undertaken to evaluate predictive performance of the model (e.g., using leave-one-out cross-validation), and I did not spot any formal attempt of model selection. In essence, the authors use a single model and rely on visualizations to spot where data and model are congruent. In all, I do not suggest that the authors should actually address the above points by adding more models and analyses, but it does lessen my enthusiasm for the manuscript. I suggest the authors put more effort in a critical evaluation of their model in the discussion, and perhaps could add something on cross-validation and model selection in the main analyses.

*-We evaluated the performance of the model by predicting trajectories, including 90% confidence intervals, based on data up to, but not including, those two weeks. We then determine whether the true trajectory for those two weeks is within the 90% confidence interval (Figure 4). This is a more relevant statistic than using standard cross-validation methods, which essentially requires dividing your data into a training and validation dataset. The process is described in detail in the methods. Because the model is only interested in forward projection, the evaluation method used is appropriate since this is scoring the model against the target product. We agree that comparison with other models would be interesting, but this work focussed on a highly motivated mechanistic model structure, and the purpose of the paper is to evaluate performance of this given model.*

Specific comments/suggestions

intro, second paragraph. please add that assessment of the current situation (i.e nowcasting) is a problem in itself. Also add in the discussion how your results are affected if cases (by admission date) are only complete after some time (two weeks? is this a problem?)

*-There is only a slight issue of incompleteness in this model, whereby hospital admissions are lagged on the last two days and deaths for around 5 days. Because of the nature of the data collection, there are not substantial lags in the hospital data, and reporting delays for deaths in hospital are relatively short. So the nowcasting challenge would be to predict the two days of slight lags in admissions, which does not pose an interesting problem. For other data collection mechanisms, this may be more of an issue.*

Figure 1. I found this figure not very appealing visually, and the legend difficult to follow.

*-This figure has been tidied up and moved to the supplementary material.*

"With the foresight of formulating ...". It is of course quite convenient that delay distributions are apparently well-described by exponential distributions. It is, however, not difficult to rewrite the model from the hospital compartment (L_H) onwards in terms of delay equations. Please discuss and elaborate in the Discussion.

-We have now added the following point to the discussion: *“The structure of EpiBeds makes use of the fact that the delay distributions were approximately Erlang distributed, so that they can easily be approximated by a series of ODEs. It would be possible to instead write the model in terms of delay equations, but the ODE approach leads to significantly reduced computational cost, which is essential for a modelling product that may need to be run multiple times per week.”*

Figure 2. I found this somewhat superfluous, especially the distinction between i. and ii.

*-We have removed this figure to make the paper more concise, but retain the discussion around these different approaches since we feel this makes an important distinction.*

"The structure for the generalised ...". Please mention once that formally these are a specific class of exponential distributions, i.e. Erlang distributions.

*-We mentioned this multiple times in the methods section, but have now added it to the results section as well. NB. We appreciate that the original version was overly long and not as well structured as it could have been, and so understand that this could have been missed.*

"This level of accuracy is sufficient since ...". This strikes me as an unwarranted claim. Please remoce or explain in detail.

*-We have deleted the statement*

Section 2.3.1. Please provide rationale WHY most parameters are fixed while only a small number is estimated. Especially, as in the Bayesian context you have the flexibility to add (weakly) informative priors. Why did you include a highly informative prior for p_D but not for the other p parameters?

-We now explain why we chose an informative prior for p_D (see response to Reviewer 1). *The other p parameters could not be estimated reliably prior to the modelling, because of strong biases in the individual-level data we had available. During the pandemic, various estimates for these have been calculated, but at the time of model construction no reliable estimates existed, so we wanted the model to be able to estimate these accurately. We agree that prior distributions for the other parameters, especially length of stay, could have been included in the model, but opted to keep these as fixed to reduce the computational cost of the model. With the modelling being run multiple times each week, it was important to have something that ran quickly and without needing high-performance computing. The drawback of this is that uncertainty in forward projections is smaller than what a model with prior distributions for the length of stay would generate.*

Figure 4, bottom left. There is a systematic deviation from the posterior median. Explain in intuitive terms? Also, are bands actual Bayesian prediction intervals, or CrIs?

*-These are prediction intervals as they are modelling both uncertainty in the parameter values describing the system dynamics (and hence the central trend of the data) and the overdispersion of the data generating process. The systematic deviation arises as the data streams are starting to be inconsistent with the model structure. Hence after this point we started fitting to a subset of the data streams in order to reparameterise the relationship. This was described in Section 2.3.3 “This is driven by multiple factors including: inconsistencies between data streams at the start of the first wave; a large shift in the age distribution of admissions from frailer older people in the spring to younger people with low mortality risk in the summer; and changes in treatment which likely altered outcome probabilities.”*
